# Risk factors associated with false negative rate of sentinel lymph node biopsy in endometrial cancer: a systematic review and meta-analysis

**DOI:** 10.3389/fonc.2024.1391267

**Published:** 2024-04-03

**Authors:** Meng-si Fan, Ke-xin Qiu, Dong-yue Wang, Hao Wang, Wei-wei Zhang, Li Yan

**Affiliations:** ^1^ Department of Gynecology, Shandong Provincial Qianfoshan Hospital, Shandong Second Medical University, Key Laboratory of Laparoscopic Technology, The First Affiliated Hospital of Shandong First Medical University, Jinan, China; ^2^ School of Clinical Medicine, Shandong First Medical University, Jinan, China; ^3^ Department of Gynecology, Tengzhou Maternal and Child Health Hospital, Tengzhou, Shandong, China; ^4^ Department of Gynecology, The First Affiliated Hospital of Shandong First Medical University & Shandong Provincial Qianfoshan Hospital, Jinan, China

**Keywords:** sentinel lymph node, sentinel lymph node biopsy, endometrial neoplasms, endometrial cancer, meta-analysis

## Abstract

**Objective:**

Currently, sentinel lymph node biopsy (SLNB) is increasingly used in endometrial cancer, but the rate of missed metastatic lymph nodes compared to systemic lymph node dissection has been a concern. We conducted a systematic review and meta-analysis to evaluate the false negative rate (FNR) of SLNB in patients with endometrial cancer and to explore the risk factors associated with this FNR.

**Data sources:**

Three databases (PubMed, Embase, Web of Science) were searched from initial database build to January 2023 by two independent reviewers.

**Research eligibility criteria:**

Studies were included if they included 10 or more women diagnosed with International Federation of Gynecology and Obstetrics (FIGO) stage I or higher endometrial cancer, the study technique used sentinel lymph node localization biopsy, and the reported outcome metrics included false negative and/or FNR.

**Study appraisal and synthesis methods:**

Two authors independently reviewed the abstracts and full articles. The FNR and factors associated with FNR were synthesized through random-effects meta-analyses and meta-regression.

**The results:**

We identified 62 eligible studies. The overall FNR for the 62 articles was 4% (95% CL 3-5).There was no significant difference in the FNR in patients with high-risk endometrial cancer compared to patients with low-risk endometrial cancer. There was no difference in the FNR for whether frozen sections were used intraoperatively. The type of dye used intraoperatively (indocyanine green/blue dye) were not significantly associated with the false negative rate. Cervical injection reduced the FNR compared with alternative injection techniques. Indocyanine green reduced the FNR compared with alternative Tc-99m. Postoperative pathologic ultrastaging reduced the FNR.

**Conclusions:**

Alternative injection techniques (other than the cervix), Tc-99m dye tracer, and the absence of postoperative pathologic ultrastaging are risk factors for a high FNR in endometrial cancer patients who undergo SLNB; therefore, we should be vigilant for missed diagnosis of metastatic lymph nodes after SLNB in such populations.

**Systematic review registration:**

http://www.crd.york.ac.uk/PROSPERO/, identifier CRD42023433637.

## Introduction

Endometrial cancer is one of the three major malignant tumors of the female reproductive tract. In recent years, EC, with its increasing incidence and mortality worldwide, has become the most common gynecologic cancer in high-income countries (1, 2), posing a serious threat to public health worldwide.

Surgery is currently the main modality for the treatment of endometrial cancer. Despite the low rate of metastasis in patients with early endometrial cancer, the standard of care still includes complete or elective pelvic and para-aortic lymph node dissection for proper staging, which is the most important prognostic factor (3, 4). Benedetti Panici et al. and the ASTEC trial have demonstrated that lymphadenectomy has a staging role and does not improve overall survival in low risk populations (5, 6). In addition, the number of lymph nodes removed during staging is associated with potential side effects (7). Since 2016, SLNB has been introduced as an alternative to lymph node dissection for surgical staging (8). The majority of SLNs were located in the pelvic area, with the external iliac vessels being the most common area (9). Theoretically, sentinel lymph node (SLN) should reflect the status of the entire nodal basin. If there is no metastasis in the sentinel lymph nodes, the likelihood of metastasis to other lymph nodes in the pelvis is extremely low. At present, lymphadenectomy is primarily used for staging and should be considered in women with high- risk factors; however, sentinel lymph node biopsy is an acceptable alternative to systematic lymphadenectomy in early- stage endometrial cancer (10). The recent guidelines from the European Society of Gynecological Oncology-European Society for Radiotherapy and Oncology-European Society of Pathology (ESGO-ESTRO-ESP) have expressed unanimous consensus to consider SLN biopsy for staging purposes in patients with low risk, intermediate risk, and high risk of endometrial cancer (11). Retrospective studies showed similar prognosis of patients after full lymphadenectomy and sentinel lymph node biopsy only (12–14). In addition, compared to complete lymph node dissection, this procedure avoids complications associated with systemic lymph node dissection, such as neurovascular damage, lymphedema and lymphoid cyst formation (15, 16).

Although SLN localization has many advantages, the rate of missed metastatic lymph nodes compared to systemic lymph node dissection has been a concern. In particular, the value of sentinel lymph node biopsy in patients with high-risk types of endometrial cancer for its application remains controversial (17). At the same time, the presence of micrometastases in some SLNs, which are undiagnosed by conventional histology (18), has recently increased the rate of missed metastatic lymph nodes. In recent years, scholars in several studies have reported false negatives in endometrial cancer using SLNB (19–21). Scholars in current studies point out that in endometrial cancer, SLN biopsy combined with standard algorithms and ultrastaging has been shown to significantly reduce the false negative rate and improve sensitivity and negative predictive value (22). However, even when different intraoperative algorithms for sentinel lymph nodes are followed and pathologic ultrastaging is used, some patients with lymph node metastases are still missed to the point of compromising postoperative adjuvant therapy and presenting a poor prognosis.

Although there is a proliferation of articles examining false negative rates of sentinel lymph node biopsies for endometrial cancer, they are mostly limited to reports of individual rates of false negative rates. Few studies have been performed on the risk factors affecting the false negative rate. Therefore, the aim of this meta-analysis was to evaluate the false negative rate of SLNB performed in patients with endometrial cancer, as well as to explore the risk factors affecting the FNR, to rationally prevent and minimize the incidence of the false negative rate and to evaluate whether sentinel sentinel lymph node biopsy is a feasible technological option in patients with high-risk endometrial cancer.

## Methods

### Information sources and search strategy

We searched three databases (PubMed, Embase, and Web of Science) for articles from the time of their creation to January 2023. The following Medical Subject Headings were used: “Sentinel Lymph Node,” “Sentinel Lymph Node Biopsy,” and “Endometrial Neoplasms” (**Table 1**). The titles and abstracts of each retrieved article were reviewed to confirm that the article reported false negative sentinel lymph node biopsies in patients with endometrial cancer, and those articles that met the criteria for inclusion were retrieved in full and further reviewed for literature supplementation using references cited in articles that met the criteria for inclusion. Details of the review protocol were registered in PROSPERO, number CRD42023433637.

**Table 1:** Search terms.

Pubmed	Embase	Web of seince
1.(((((((("Sentinel Lymph Node"[Mesh]) OR (Lymph Node, Sentinel[Title/Abstract])) OR (Lymph Nodes, Sentinel[Title/Abstract])) OR (Sentinel Lymph Nodes[Title/Abstract])) OR (Sentinal Node[Title/Abstract])) OR (Node, Sentinal[Title/Abstract])) OR (Nodes, Sentinal[Title/Abstract])) OR (Sentinal Nodes[Title/Abstract])) OR ((((((((("Sentinel Lymph Node Biopsy"[Mesh]) OR (Lymph Node Biopsy, Sentinel[Title/Abstract])) OR (Biopsy, Sentinel Lymph Node[Title/Abstract])) OR (sentinel axillary lymph node biopsy[Title/Abstract])) OR (sentinel lymph node biopsies[Title/Abstract])) OR (sentinel lymphatic node biopsy[Title/Abstract])) OR (sentinel nodal biopsy[Title/Abstract])) OR (sentinel node biopsy[Title/Abstract])) OR (SLN biopsy[Title/Abstract])) 2.(((((((((((((((((((((((((("Endometrial Neoplasms"[Mesh]) OR (endometrium tumor[Title/Abstract])) OR (endometrial neoplasms[Title/Abstract])) OR (endometrial tumor[Title/Abstract])) OR (endometrial tumour[Title/Abstract])) OR (endometrioma[Title/Abstract])) OR (endometrium tumour[Title/Abstract])) OR (Endometrial Neoplasm[Title/Abstract])) OR (Neoplasm, Endometrial[Title/Abstract])) OR (Neoplasms, Endometrial[Title/Abstract])) OR (Endometrial Carcinoma[Title/Abstract])) OR (Carcinoma, Endometrial[Title/Abstract])) OR (Carcinomas, Endometrial[Title/Abstract])) OR (Endometrial Carcinomas[Title/Abstract])) OR (Endometrial Cancer[Title/Abstract])) OR (Cancer, Endometrial[Title/Abstract])) OR (Cancers, Endometrial[Title/Abstract])) OR (Endometrial Cancers[Title/Abstract])) OR (Endometrium Cancer[Title/Abstract])) OR (Cancer, Endometrium[Title/Abstract])) OR (Cancers, Endometrium[Title/Abstract])) OR (Cancer of the Endometrium[Title/Abstract])) OR (Carcinoma of Endometrium[Title/Abstract])) OR (Endometrium Carcinoma[Title/Abstract])) OR (Endometrium Carcinomas[Title/Abstract])) OR (Cancer of Endometrium[Title/Abstract])) OR (Endometrium Cancers[Title/Abstract]) 3.1AND2	1.'sentinel lymph node'/exp OR 'sentinel lymph node' OR (('sentinel'/exp OR sentinel) AND ('lymph'/exp OR lymph) AND node) OR 'lymph node, sentinel' OR (('lymph'/exp OR lymph) AND node, AND ('sentinel'/exp OR sentinel)) OR 'lymph nodes, sentinel' OR (('lymph'/exp OR lymph) AND nodes, AND ('sentinel'/exp OR sentinel)) OR 'sentinel lymph nodes' OR (('sentinel'/exp OR sentinel) AND ('lymph'/exp OR lymph) AND nodes) OR 'sentinal node' OR (sentinal AND node) OR 'node, sentinal' OR (node, AND sentinal) OR 'nodes, sentinal' OR (nodes, AND sentinal) OR 'sentinal nodes' OR (sentinal AND nodes) 2.'sentinel lymph node biopsy'/exp OR 'sentinel lymph node biopsy' OR (('sentinel'/exp OR sentinel) AND ('lymph'/exp OR lymph) AND node AND ('biopsy'/exp OR biopsy)) OR 'lymph node biopsy, sentinel'/exp OR 'lymph node biopsy, sentinel' OR (('lymph'/exp OR lymph) AND node AND ('biopsy,'/exp OR biopsy,) AND ('sentinel'/exp OR sentinel)) OR 'biopsy, sentinel lymph node'/exp OR 'biopsy, sentinel lymph node' OR (('biopsy,'/exp OR biopsy,) AND ('sentinel'/exp OR sentinel) AND ('lymph'/exp OR lymph) AND node) OR 'sentinel axillary lymph node biopsy'/exp OR 'sentinel axillary lymph node biopsy' OR (('sentinel'/exp OR sentinel) AND axillary AND ('lymph'/exp OR lymph) AND node AND ('biopsy'/exp OR biopsy)) OR 'sentinel lymph node biopsies'/exp OR 'sentinel lymph node biopsies' OR (('sentinel'/exp OR sentinel) AND ('lymph'/exp OR lymph) AND node AND ('biopsies'/exp OR biopsies)) OR 'sentinel lymphatic node biopsy'/exp OR 'sentinel lymphatic node biopsy' OR (('sentinel'/exp OR sentinel) AND ('lymphatic'/exp OR lymphatic) AND node AND ('biopsy'/exp OR biopsy)) OR 'sentinel nodal biopsy'/exp OR 'sentinel nodal biopsy' OR (('sentinel'/exp OR sentinel) AND nodal AND ('biopsy'/exp OR biopsy)) OR 'sentinel node biopsy'/exp OR 'sentinel node biopsy' OR (('sentinel'/exp OR sentinel) AND node AND ('biopsy'/exp OR biopsy)) OR 'sln biopsy'/exp OR 'sln biopsy' OR (sln AND ('biopsy'/exp OR biopsy)) 3.'endometrium tumor'/exp OR 'endometrium tumor' OR (('endometrium'/exp OR endometrium) AND ('tumor'/exp OR tumor)) OR 'endometrial neoplasms'/exp OR 'endometrial neoplasms' OR (endometrial AND ('neoplasms'/exp OR neoplasms)) OR 'endometrial tumor'/exp OR 'endometrial tumor' OR (endometrial AND ('tumor'/exp OR tumor)) OR 'endometrial tumour'/exp OR 'endometrial tumour' OR (endometrial AND ('tumour'/exp OR tumour)) OR 'endometrioma'/exp OR endometrioma OR 'endometrium tumour'/exp OR 'endometrium tumour' OR (('endometrium'/exp OR endometrium) AND ('tumour'/exp OR tumour)) OR 'endometrial neoplasm' OR (endometrial AND ('neoplasm'/exp OR neoplasm)) OR 'neoplasm, endometrial' OR (('neoplasm,'/exp OR neoplasm,) AND endometrial) OR 'neoplasms, endometrial' OR (('neoplasms,'/exp OR neoplasms,) AND endometrial) OR 'endometrial carcinoma'/exp OR 'endometrial carcinoma' OR (endometrial AND ('carcinoma'/exp OR carcinoma)) OR 'carcinoma, endometrial' OR (('carcinoma,'/exp OR carcinoma,) AND endometrial) OR 'carcinomas, endometrial' OR (carcinomas, AND endometrial) OR 'endometrial carcinomas' OR (endometrial AND carcinomas) OR 'endometrial cancer'/exp OR 'endometrial cancer' OR (endometrial AND ('cancer'/exp OR cancer)) OR 'cancer, endometrial' OR (('cancer,'/exp OR cancer,) AND endometrial) OR 'cancers, endometrial' OR (('cancers,'/exp OR cancers,) AND endometrial) OR 'endometrial cancers' OR (endometrial AND ('cancers'/exp OR cancers)) OR 'endometrium cancer'/exp OR 'endometrium cancer' OR (('endometrium'/exp OR endometrium) AND ('cancer'/exp OR cancer)) OR 'cancer, endometrium'/exp OR 'cancer, endometrium' OR (('cancer,'/exp OR cancer,) AND ('endometrium'/exp OR endometrium)) OR 'cancers, endometrium' OR (('cancers,'/exp OR cancers,) AND ('endometrium'/exp OR endometrium)) OR 'cancer of the endometrium' OR (('cancer'/exp OR cancer) AND of AND the AND ('endometrium'/exp OR endometrium)) OR 'carcinoma of endometrium' OR (('carcinoma'/exp OR carcinoma) AND of AND ('endometrium'/exp OR endometrium)) OR 'endometrium carcinoma'/exp OR 'endometrium carcinoma' OR (('endometrium'/exp OR endometrium) AND ('carcinoma'/exp OR carcinoma)) OR 'endometrium carcinomas' OR (('endometrium'/exp OR endometrium) AND carcinomas) OR 'cancer of endometrium' OR (('cancer'/exp OR cancer) AND of AND ('endometrium'/exp OR endometrium)) OR 'endometrium cancers' OR (('endometrium'/exp OR endometrium) AND ('cancers'/exp OR cancers)) 4.(1OR2)AND3	1.(((((((TS=(Sentinel Lymph Node)) OR AB=(Lymph Node, Sentinel)) OR AB=(Lymph Nodes, Sentinel)) OR AB=(Sentinel Lymph Nodes)) OR AB=(Sentinal Node)) OR AB=(Node, Sentinal)) OR AB=(Nodes, Sentinal)) OR AB=(Sentinal Nodes)(14163) 2. ((((((((TS=(Sentinel Lymph Node Biopsy)) OR AB=(Lymph Node Biopsy, Sentinel)) OR AB=(Biopsy, Sentinel Lymph Node)) OR AB=(sentinel axillary lymph node biopsy)) OR AB=(sentinel lymph node biopsies)) OR AB=(sentinel lymphatic node biopsy)) OR AB=(sentinel nodal biopsy)) OR AB=(sentinel node biopsy)) OR AB=(SLN biopsy)(9794) 3.((((((((((((((((((((((((((TS=(Endometrial Neoplasms)) OR AB=(endometrium tumor)) OR AB=(endometrial neoplasms)) OR AB=(endometrial tumor)) OR AB=(endometrial tumour)) OR AB=(endometrioma)) OR AB=(endometrium tumour)) OR AB=(Endometrial Neoplasm)) OR AB=(Neoplasm, Endometrial)) OR AB=(Neoplasms, Endometrial)) OR AB=(Endometrial Carcinoma)) OR AB=(Carcinoma, Endometrial)) OR AB=(Carcinomas, Endometrial)) OR AB=(Endometrial Carcinomas)) OR AB=(Endometrial Cancer)) OR AB=(Cancer, Endometrial)) OR AB=(Cancers, Endometrial)) OR AB=(Endometrial Cancers)) OR AB=(Endometrium Cancer)) OR AB=(Cancer, Endometrium)) OR AB=(Cancers, Endometrium)) OR AB=(Cancer of the Endometrium)) OR AB=(Carcinoma of Endometrium)) OR AB=(Endometrium Carcinoma)) OR AB=(Endometrium Carcinomas)) OR AB=(Cancer of Endometrium)) OR AB=(Endometrium Cancers)(20884) 4.(1OR2)AND3

### Eligibility criteria

Inclusion criteria included 10 or more women diagnosed with FIGO stage 1 or higher endometrial cancer; study technique using sentinel lymph node localization biopsy; after sentinel lymph node dissection was performed pelvic lymphadenectomy with or without paraaortic lymphadenectomy; and reported outcome metrics including but not limited to false negative/false negative rates. The exclusion criteria were as follows: articles that did not meet the inclusion criteria; secondary lesions and repeat populations or nonoriginal studies (e.g., systematic reviews); and narrative reviews, letters, editorials, conferences, and abstracts. If duplicate data sets were encountered, the most recent or informative study was included in the analysis.

### Study selection

We managed the literature screening using EndNote X 20, and after removing duplicates, 2 researchers browsed the titles and abstracts by mutual blindness, performed preliminary screening of the literature according to the inclusion and exclusion criteria, excluded the literature that did not meet the inclusion criteria, and performed full-text browsing for articles that met the inclusion criteria, while cross-checking them afterward and exchanging opinions through discussion or seeking third-party opinions in case of disagreement. The cross-checking was followed by cross-checking, and disagreements were resolved by discussing and exchanging opinions or seeking opinions from third parties. Subsequently, a standardized form was used to extract information from the included literature, including the authors, year, sample size, tumor histology type, surgical route, and other basic information.

### Data extraction

Data were extracted by two independent reviewers using a standardized form. Extracted data included authors, year of publication, sample size, study method, tumor histology type, tracer, injection site, surgical route, age, BMI, tumor risk grade, intraoperative frozen sections, pathological ultrastaging, and quality assessment items. The surgical route included (robotic, laparoscopic, open), injection site included (cervical vs. intrauterine), tracer included (indocyanine green, blue dye, Tc-99 m), intraoperative frozen section and pathological ultrastaging included (yes/no).

There are many methods of SLN ultrastaging, and the one currently used is the Memorial Sloan Kettering Cancer Center (MSKCC) superstaging method: first, paraffin sections are routinely stained for H&E, and if the results are negative, two consecutive 5-μm-thick sections (one for H&E and one for cytokeratin AE1/AE3) at 50-μm intervals from each paraffin block are rowed, with one of the levels providing another section as a negative control for immunohistochemistry (23). Ultrastaging was defined in this meta-analysis asl any additional treatment of the sentinel lymph nodes beyond routine lymph node evaluation, usually including additional sectioning and hematoxylin and eosin (H&E) staining of the SLN; ultrastaging was considered in all cases where immunohistochemistry was used. Intraoperative frozen section was defined as postoperative pathological examination of sentinel lymph node sections after rapid frozen sectioning. The false negative rate was defined as metastatic patients without a positive SLN divided by all metastatic patients (false negative tests/false negative + true positive tests) (24). High-risk tumors were defined as fulfilling one of the following conditions: high-grade tumor (endometrioid grade 3 and nonendometrioid histologies: serous, clear cell, or carcinosarcoma), deep myometrial invasion (MI) (≥50%), or the presence of angiolymphatic invasion (LVSI) (25). When different definitions were used for reporting outcomes, we recalculated the original study results according to our proposed definitions.

### Assessment of risk of bias

Two reviewers independently assessed the risk of bias for inclusion in the study using the QUADAS-2 tool. Differences were resolved through review of the original articles. The risk of bias was assessed in four domains: patient selection, index test, reference standard, and flow and timing. Bias in the patient selection domain could occur if recruitment was not consecutive, not random, or if inappropriate exclusion criteria were used. Bias in the index test domain could occur if the interpretation of the results of the test to be evaluated is performed with knowledge of the results of the gold standard test, or if the test threshold is chosen to optimize sensitivity and/or specificity. Bias in the reference standard domain could occur if the interpretation of the gold standard results is performed with knowledge of the results of the test to be evaluated. Bias in the flow and timing domain could occur If only a certain percentage of the study group received the gold standard, or if some patients received a different gold standard, or if not all cases included in the study were included in the analysis. A study was judged to be at “low”, “unclear”, or “high” risk of bias in each domain, based on a set of signaling questions for each domain. If all signaling questions for a domain were answered “yes” then the risk of bias was judged as “low” for that domain; similarly, if any signaling question was answered “no” then the risk of bias was judged to be “high” for that domain. The “unclear” category was used when there was insufficient data to allow the judgment. For clinical applicability, only the first three components (patient selection, index test and reference standard) are evaluated, and the determination method is the same as that of the risk of bias, which is based on the degree of matching with the evaluation question, and is also graded according to the levels of “high”, “low”, and “unclear”.

### Data synthesis

We used the ‘meta’ package v4.15-1 in R v3.5.0 for statistical analysis. We evaluated statistical heterogeneity with the use of thestatistic and defined heterogeneity as notable when >50%. We calculated the overall false negative rate based on the data provided in the original paper, and we performed a meta-analysis of the false negative rate using a random-effects model. We used stratified meta-analysis and meta-regression to explore the impact of patient (tracer, injection site, tumor risk class, intraoperative frozen section, pathological ultrastaging) characteristics on the combined outcomes. When studies reported outcomes for multiple subgroups (e.g., comparing injection sites), we included the overall false negative rate in the main meta-analysis and the subgroup false negative rate in the stratified meta-analysis and meta-regression. A value of p < 0.05 was considered significant.

## Results

### Study selection

**Figure 1:**
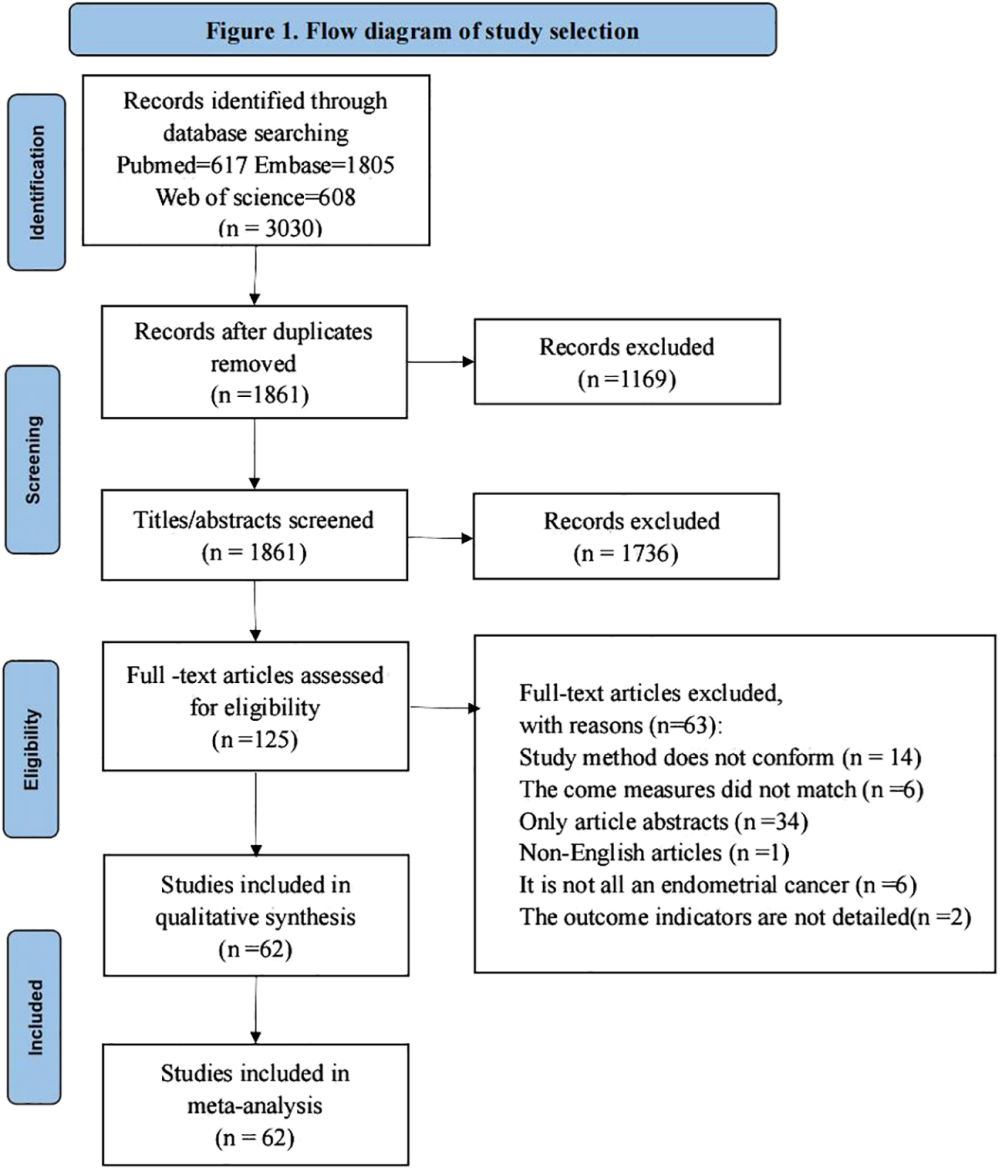
Flow diagram of study selection.

The study obtained a total of 3030 relevant studies by searching three databases ((PubMed, Embase, Web of Science), 1861 remaining studies after excluding duplicate items, 125 remaining relevant studies after reading the titles and abstracts of the literature to exclude studies not relevant to the study, and 125 full-text studies after reading to exclude studies not consistent with the purpose of the study. Finally, a total of 62 studies were included for qualitative synthesis and meta-analysis, and the literature screening process and results are shown in **Figure 1**.

### Study characteristics

Of the 62 articles included in this study (19–21, 25–83), we used only those with endometrial cancer patients who had at least pelvic lymph node dissection after sentinel lymph node dissection as the study population. The total included sample size was 6304 cases, the maximum sample size for a single study was 414 cases, and the minimum sample size for a single study was 17 cases; pelvic ± para-aortic lymphadenectomy biopsy was the most commonly used reference standard; all literature reported relevant study outcome indicators; specific description of the included literature (**Table 2**). Of the 62 articles, 35 (56.5%) were prospective studies, 22 (35.5%) were retrospective studies, and the remaining 5 (8%) articles did not clearly account for the study methods.

**Table 2:** Characteristics of included studies.

Author	Year	Study Size	Study Method	Cancer Histology	Tracer Used	Injection Site	Route of Surgery	Reference Standard	Pathology Assessment
E. Barranger	2004	17	Prospective	All	Patent Blue and Tc-99m	Cervical	Laparoscopic	Systematic pelvic lymphadenectomy, para-aortic lymphadenectomy was performed when a para-aortic SN was detected or when positive SNs were found by intraoperative histological examination	H&E, IHC、ultrastaging
H. Niikura	2004	28	NR	All	Tc-99m	Uterine	Open	Pelvic and paraaoPrtic lymphadenectomy	H&E, IHC、ultrastaging
C. Altgassen	2007	25	Prospective	All	Blue dye	Uterine	Open	Depending on tumor size (>2 cm), grading (>G1) and invasion (>Ia) a complete pelvic lymphadenectomy was performed. A para-aortic lymphadenectomy followed if the general state of the patient was considered to be sufficient by the gynecologist and the anesthesiologist intra-operatively	H&E
L. A. Lopes	2007	40	NR	All	Patent Blue	Uterine	Open	Bilateral para-aortic and pelvic lymphadenectomies	H&E, IHC、ultrastaging
A. S. Bats	2008	43	Prospective	All	Tc-99m and blue dye	Cervical	Laparoscopic	Pelvic lymphadenectomy was performed with or without para-aortic lymphadenectomy	H&E, IHC、ultrastaging
Barranger E	2009	33	Prospective	All	Tc-99m and patent blue	Cervical	Laparoscopic	Laparoscopic bilateral pelvic lymphadenectomy	H&E, IHC、ultrastaging
J. How	2012	100	Prospective	All	Tc-99m and patent blue	Cervical	Robotic	Complete pelvic lymphadenectomy in all cases regardless of surgical pathology diagnosis. If the patient had a pre-operative type II endometrial cancer (clear cell, serous,adeno-squamous) or grade 2 or 3 endometrioid carcinomas, or carcinosarcoma, positive SLN on intra-operative frozen section, or grossly enlarged pelvic LNs suspicious for malignancy, the surgeon would continue with a para-aortic lymphadenectomy	H&E, IHC、ultrastaging
E. Solima	2012	59	Prospective	All	Tc-99m	Uterine	Open or Laparoscopic	Patients with one of the following criteria underwent systematic pelvic and paraaortic lymphadenectomy: 1) endometrioid adenocarcinoma with intraoperative staging equal to or higher than IBG2 (FIGO 1988); and 2) clear cell or serous carcinoma. All surgeries were performed by 4 senior gynecologists with proficiency in oncologic surgery, experienced in radio guided surgery in endometrial cancer.	H&E, IHC、ultrastaging
E. C. Rossi	2013	29	Prospective	All	ICG	Cervical & Uterine	Robotic	Bilateral pelvic and paraaortic lymphadenectomy	H&E 、 ultrastaging
A. Torné	2013	74	Prospective	All	Tc-99m	Uterine	Laparoscopic	Laparoscopic pelvic and paraaortic lymphadenectomy	H&E, IHC、ultrastaging
C. L. D. Cano	2014	50	Prospective	All	Tc-99m and blue dye	Cervical	Open or Laparoscopic	Pelvic and/or paraaortic lymphadenectomy	H&E 、 ultrastaging
E. Raimond	2014	156	Retrospective	All	Patent blue	Cervical	NR	Pelvic lymphadenectomy was systematically	H&E, IHC、ultrastaging
M. M. Farghali	2015	93	Retrospective	Endometrioid adenocarcinoma、Clear Cell Carcinoma 、Papillary serous carcinoma	Methylene blue dye	Uterine	Open	Pelvic and para-aortic lymphadenectomy. Extent of lymphadenectomy was decided by senior surgeon intra-operatively depending on grade of tumor, depth of invasion, size, location of endometrial carcinoma and patient`s fitness to such risky intervention	H&E, IHC、ultrastaging
G. Favero	2015	42	Prospective	Endometrioid adenocarcinoma、Serous papillary adenocarcinoma、Clear cell adenocarcinoma	Tc-99m	Uterine	Laparoscopic	Pelvic and Bpara-aortic lymphadenectomy	H&E
J. How	2015	100	Prospective	All	Tc-99m、ICGandBlue day	Cervical	Robotic	Pelvic lymphadenectomy. A para-aortic lymphadenectomy was per-formed if the patient had one or more of the following characteristics:pre-operative type II endometrial cancers (clear cell, serous, carcinosarcoma, or adenosquamous), grade 3 endometrioid carcinomas, positive SLN on intraoperative frozen section, or grossly enlarged para-aortic LNs suspicious for malignancy	H&E, IHC、ultrastaging
Naoura I	2015	180	Retrospective	All	Tc-99m and patent blue	Cervical	Open or Laparoscopic	SLN procedure completed by a systematic pelvic lymphadenectomy.Para-aortic lymphadenectomy (PAAL) was recommended for patients with positive SLN at intraoperative examination or final histology and for those with high-risk EC according to the current guidelines	H&E, IHC、ultrastaging
Sawicki S	2015	60	NR	Endometrioid 、Clear cell 、Serous	(Tc-99m and blue) dye or blue dye	cervical and Uterine	Open	Pelvic and para-aortic lymphadenectomy (with SLNB) was performed in patients with grade 3 tumors, with more than 50% myometrial invasion or cervical involvement (in the latter 2 cases, the extent of para-aortic lymph node dissection was at a surgeon’ s discretion).	H&E、ultrastaging
Touhami O	2015	39	Retrospective	Uterine Serous Carcinoma	Tc-99m and patent blue	Uervical	Open or Laparoscopic	Pelvis lymph nodes + para-aortic lymph nodes.	NR
P. Valha	2015	18	Prospective	All	Blue dye	Uterine	Open	Pelvic and para-aortic lymphadenectomies	H&E, IHC、ultrastaging
A. Buda	2016	118	Retrospective	All	Tc-99m and blue dye or blue day or ICG	Cervical	Open or Laparoscopic	Complete pelvic lymphadenectomy and in the absence of SLN mapping or unilateral mapping	H&E, IHC、ultrastaging
J. Ehrisman	2016	36	Retrospective	All	Meth-ylene blue or indocyanine green (ICG)	Cervical	Laparoscopic	Complete pelvic lymphadenectomy	H&E, IHC、ultrastaging
R. W. Holloway	2016	119	Retrospective	All	ICG	Cervical	Robotic	A pelvic lymphadenectomy or pelvic-plus-aortic lymphadenectomy Para-aortic lymphadenectomy was reserved for Grade 3 tumors/Type II histologies with any depth-of-invasion (DOI),grossly positive pelvic lymph nodes confirmed on frozen section,and low-grade tumors with middle or outer-third myometrial invasion.	H&E, IHC、ultrastaging
P. J. Paley	2016	85	Prospective	All	ICG	Cervical	Robotic	Complete pelvic and paraaortic lymphadenectomy if high risk or in the absence of SLN mapping or unilateral mapping	H&E
A. Papadia	2016	42	Retrospective	All	ICG	Cervical	Laparoscopic	PLND and/or PALND	H&E, IHC、ultrastaging
G. Baiocchi	2017	75	Prospective	All	Patent blue dye	Cervical	NR	Pelvic ± para-aortic lymphadenectomy	H&E, IHC、ultrastaging
I. Biliatis	2017	54	Prospective	All	Methylene blue or patent blue	Uterine	NR	Bilateral pelvic lymphadenectomy	H&E
F. Farzaneh	2017	30	Prospective	Endometrioid、Papillary serous	Tc-99m or( TC-99m and Blue dye)	Cervical	NR	Pelvic lymphadenectomy in all cases and para-aortic lymphadenectomy in selected cases (clear cell, papillary serous, grade 2 or 3 endometrioid adenocarcinomas, stage II).	NR
R. W. Holloway	2017	200	Prospective	All	ISB + ICG or ISB	Cervical	Robotic	Pelvic lymphadenectomy was performed in all cases. Para-aortic lymphadenectomy procedures were performed for patients with endometrioid G1 or G2 tumors and 50% or more myometrial invasion on frozen section or any G3 and type 2 histologies, and for patients with suspicious pelvic lymph nodes confirmed with metastases on frozen section.	H&E, IHC、ultrastaging
E. C. Rossi	2017	340	Prospective	All	ICG	Cervical	Robotic	Pelvic lymphadenectomy with or without para-aortic lymphadenectomy.	H&E, IHC、ultrastaging
P. T. Soliman	2017	101	Prospective	All	ICG or Blue dye or(TC-99m and Blue dye)	Cervical	Robotic or Laparoscopic	Pelvic and para-aortic lymphadenectomy	H&E, IHC、ultrastaging
E. J. Tanner	2017	52	Prospective	All	ISB or ICG	Cervical	Robotic	Pelvic and para-aortic lymph nodes	H&E, IHC、ultrastaging
S. Taskin	2017	71	Prospective	All	ICG	Cervical	Laparoscopic	Complete pelvic lymphadenectomy,paraaortic lymphadenectomy if high risk	H&E, IHC、ultrastaging
O. Touhami	2017	128	Retrospective	All	Blue dye、Technetium-99、Indocyanine green、Blue dye + technetium-99、Indocyanine green + technetium-99	Cervical	Laparoscopic or Robotic or Open	Pelvic lymphadenectomy,the indication and extent of the para-aortic lymphadenectomy (PAL) was left at the discretion of the surgeon.	H&E, IHC、ultrastaging
N. Body	2018	119	Retrospective	All	ICG	Cervical	Laparoscopic or Robotic or Open	All patients underwent total hysterectomy and bilateral salpingo-oophorectomy and a complete pelvic lymph node dissection following SLN mapping. Paraaortic node dissection was performed at the surgeon's discretion.	H&E, IHC、ultrastaging
K. J. Eoh	2018	50	NR	All	ICG	Uterine or Cervical	Laparoscopic	Systematic bilateral pelvic lymph node	H&E
A. Rajanbabu	2018	69	Prospective	All	ICG	Cervical	Robotic	Pelvic and paraaortic LND was done based on pre-operative risk factors (endometrial biopsy result and MRI staging).	H&E
C. Shimada	2018	57	Retrospective	All	TC-99m or/and ICG	Cervical	Laparoscopic or Open	Lymphadenectomy, the extent of lymphadenectomy was at the discretion of the attending surgeon.	H&E, IHC、ultrastaging
Tanaka T	2018	211	NR	All	TC-99m or IDG or ICG	Cervical	Laparoscopic or Open	All of the patients underwent laparoscopic or abdominal hysterectomy, bilateral salpingo-oophorectomy and an SLN biopsy with or without PLND and paraaortic lymph node dissection (PAND).	H&E
S. Togami	2018	113	Prospective	All	ICG	Cervical or Uterine	Laparoscopic or Robotic or Open	Complete pelvic lymphadenectomy,paraaortic lymphadenectomy if high risk	H&E
F. J. Backes	2019	184	Prospective	All	ISB and ICG	Cervical	Robotic	SLN biopsy was followed by complete pelvic lymphadenectomy (aortic lymphadenectomy at the discretion of the surgeon).	H&E, IHC、ultrastaging
J. A. Kennard	2019	414	Retrospective	All	ISB and ICG	Cervical	Robotic	Completion pelvic lymphadenectomy was performed in all patients during this time of this study for quality assurance to determine false negative rates (FNR) for surgeons in the group.	H&E, IHC、ultrastaging
J. Persson	2019	257	Prospective	All	ICG	Cervical	Robotic	Pelvic and infrarenal para-aortic lymphadenectomy	H&E, IHC、ultrastaging
S. Taşkin	2019	286	Retrospective	All	ICG or blue dye	Cervical	Laparoscopic or Robotic or Open	Pelvic ± para-aortic lymphadenectomy	H&E, IHC、ultrastaging
T. Wang	2019	98	Retrospective	All	ICG	Cervical	Laparoscopic	Pelvic ± para-aortic lymphadenectomy	H&E, IHC、ultrastaging
L. Ye	2019	131	Prospective	All	ICG	Cervical	Laparoscopic	Complete bilateral lymphadenectomy was then performed in all patients. Patients with high-risk histologies(grade 3 endometrioid, carcinosarcoma, serous, clear cell, or undifferentiated carcinoma) underwent simultaneous paraaortic lymphadenectomy to the inferior mesenteric artery and lymentectomy.	H&E, IHC、ultrastaging
J. Zuo	2019	115	Prospective	Endometrioid	Carbon nanoparticle	Cervical or Uterine	Laparoscopic	Para-aortic lymph node sampling procedures were performed for low-risk patient (Based on the MRI result: primary tumor is less than 2 cm in diameter, less than 50% myometrial invasion, and the pathology is non-poorly differentiated carcinoma), while the para-aortic lymphadenectomy was performed for non-low risk patient.	H&E, IHC、ultrastaging
Ş. Gezer	2020	81	Prospective	All	TC-99m	Cervical or Uterine	Open	Pelvic and paraaortic lymphadenectomy procedures	H&E, IHC、ultrastaging
F. Martinelli	2020	208	Retrospective	All	ICG or TC-99m	Uterine	Laparoscopic	Lymphadenectomy (pelvic ± aortic)	H&E, IHC、ultrastaging
M. Renz	2020	90	Retrospective	All	ICG	Cervical	NR	Complete pelvic lymphadenectomy,paraaortic lymphadenectomy if high risk	H&E
V. S	2020	35	Prospective	All	TC-99m	Cervical	Open	Complete pelvic and lower para-aortic lymphadenectomy	H&E
M. A. Angeles	2021	102	Prospective	All	TC-99m	Uterine	Laparoscopic	Systematic pelvic and paraaortic lymphadenectomy	H&E, IHC、ultrastaging
E. Curcio	2021	44	Retrospective	All	ICG	Cervical	Robotic	Systematic pelvic LND was performed in case of negative bilateral mapping, and for tumors >2 cm in greatest dimension or invading >50% of the myometrium. Pelvic and paraaortic LND up to the level of the renal veins was attempted for all grade 3 tumors, uterine serous cancer, clear cell carcinoma,and carcinosarcoma.	H&E, IHC、ultrastaging
M. C. Cusimano	2021	156	Prospective	All	ICG	Cervical	Laparoscopic or Robotic	Grade 2 endometrioid EC required bilateral PLND,and high-grade EC required bilateral PLND and PALND	H&E, IHC、ultrastaging
S. Liang	2021	90	Prospective	All	ICG or CNPs or ICG + CNPs	Cervical	Laparoscopic or Open	Systemic pelvic lymphadenectomy was performed. Para‐aortic lymphadenectomy was performed at the surgeon's discretion	H&E
V. G. Pineda	2021	88	Retrospective	All	ICG+Tc99 or Tc99+Blue dye or ICG	Cervical	Laparoscopic	Complete pelvic and paraaortic lymphadenectomy	H&E, IHC、ultrastaging
N. Sánchez-Izquierdo	2021	52	Retrospective	All	ICG and TC-99m	Uterine	Laparoscopic	Pelvic and paraaortic lymphadenectomy	H&E, IHC、ultrastaging
S. Somashekhar	2021	100	Prospective	All	ICG	Cervical	Robotic	Complete pelvic and para-aortic node dissection	ultrastaging
Q. Wang	2021	92	Retrospective	All	ICG	(Cervical and Uterine)or Cervial	Laparoscopic	Pelvic lymphadenectomy with or without infrarenal para‐aortic lymphadenectomy	H&E, IHC、ultrastaging
D. Altin	2022	128	Retrospective	All	MB or ICG	Cervical	Laparoscopic or Open or Robotic	Pelvic ± paraaortic lymphadenectomy	H&E, IHC、ultrastaging
M. Gedgaudaite	2022	90	Prospective	All	ICG	Cervical	Laparoscopic	LND	NR
A. Torrent	2022	48	Prospective	All	ICG and TC-99m	(Cervical and Uterine)or Cervial	NR	Pelvic ± paraaortic lymphadenectomy	H&E, IHC、ultrastaging
Y. Xue	2022	159	Retrospective	All	ICG	Cervical	Laparoscopic	Systematic lymphadenectomy	H&E, IHC、ultrastaging

H&E, Hematoxylin and eosin; IHC, Immunohistochemistry; LND, Lymphadenectomy; NR, Not reported; SLN, Sentinel lymph node; Tc-99m, Technecium-99; ICG, Indocyanine green; ISB, Isosulfane blue; MB, Methylene blue; CNP, Carbon nanoparticle.

### Risk of bias of included studies

**Figure 2:**
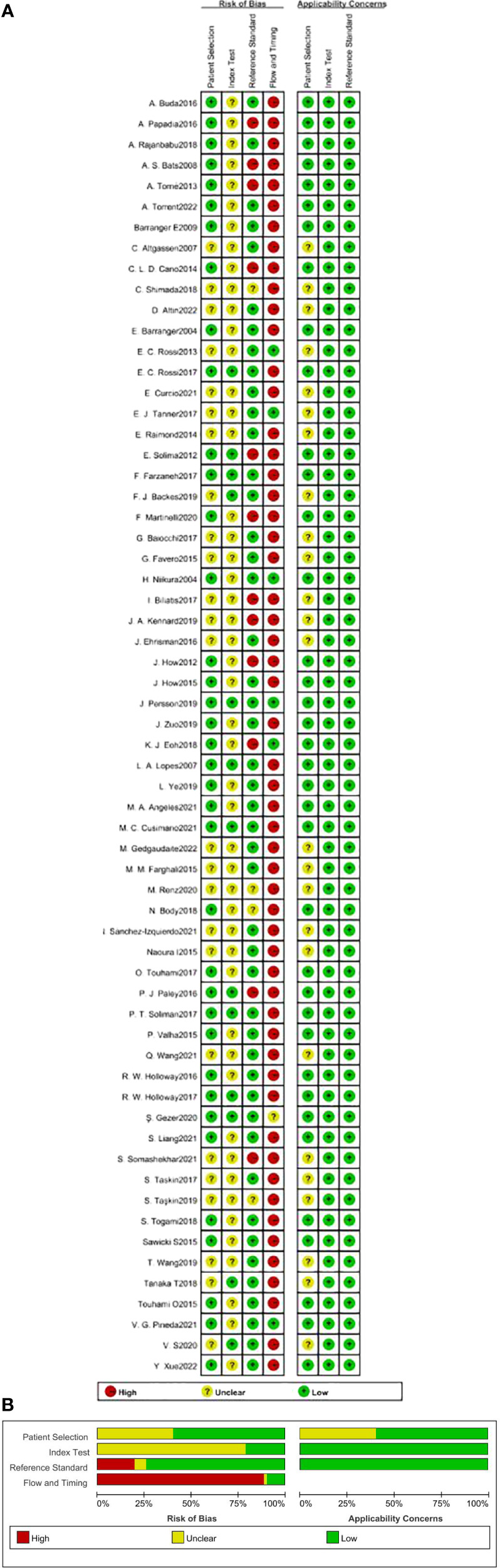
Tabular **(A)** and graphical **(B)** quality assessment of included studies.

The quality assessment of all included studies is presented in the (**Figures 2A**, **B**). The risk of bias varied across 4 domains. Most of the studies were at low risk of bias in the patient selection, index test and reference standard domains.However, 55 studies were at high risk of bias in the flow and timing domain, due to the fact that some patients received PALND in addition to PLND as a reference standard and some did not, or not included in the final analysis due to failure of sentinel lymph node visualization in some patients. All 62 studies were highly applicable to our research question across all three domains, except 25 studies containing insufficient data to make a judgement.

### Synthesis of results

The median age was reported in thirty-six studies. The study-level median age was 62.5 years (range 52-71), and the median BMI was reported in 32 studies. The study-level median BMI was 27.5 kg/m2 (range 22-38). The majority of the 62 articles 42 (67.7%) used only cervical injections, 12 (19.4%) used only uterine injections, and the remaining 8 (12.9%) used cervical and/or uterine injections. Twenty of the 62 articles (32.2%) referenced use of only ICG, 7 (11.3%) referenced use of only blue dye, 7 (11.3%) referenced use of only Tc-99 m, and the remaining 28 articles (45.2%) referenced use of multiple dyes.

### SLN false negative rate

**Figure 3:**
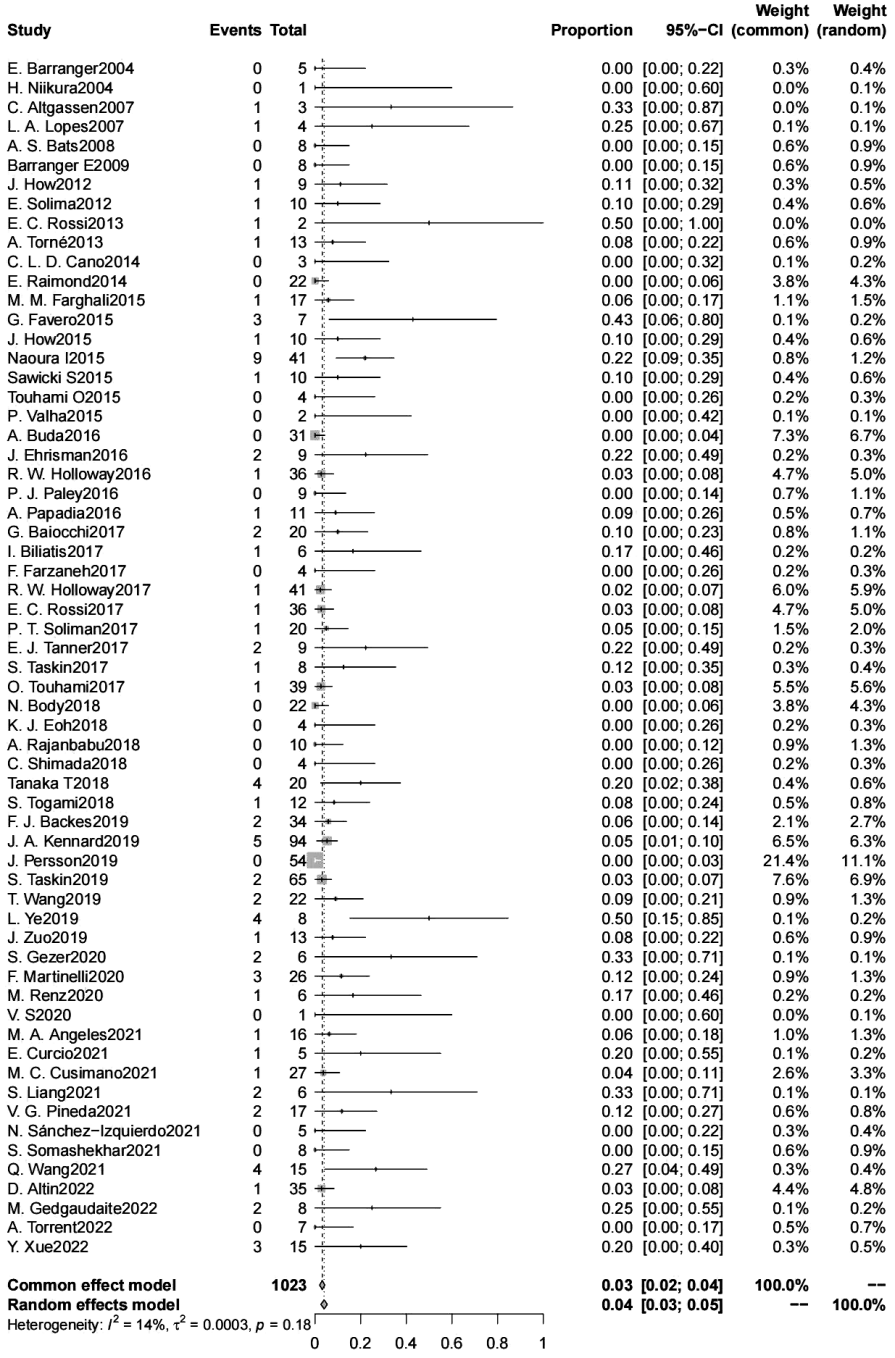
SLN false negative rate.

The false negative rate for sentinel lymph node biopsy for endometrial cancer was 4% (95% CL 3-5) (6304 patients) (**Figure 3**). There was no significant difference in the false negative rate in patients with endometrial cancer with a high-risk tumor risk grade compared with those with low-risk endometrial cancer (2600 patients); and in intraoperative frozen sections were not significantly associated with the false negative rate. The type of dye used intraoperatively (indocyanine green/blue dye) were not significantly associated with the false negative rate, the false negative rate of SLN was significantly lower for indocyanine green than for Tc-99 m (4% vs. 12%, p=0.042) (4015 patients). The false negative rate of SLN was significantly lower for cervical injections than for uterine injections (4% vs. 10%, p=0.024) (6083 patients). The use of postoperative pathologic ultrastaging was associated with a lower rate of false negative SLNs (4% vs. 11%, p=0.036) (6095 patients) (**Table 3**).

**Table 3:** Univariate Meta-Regression of SLN false negative rate and study variables.

Study Variables	Number of Patients	Overall Detection% (95% CI)	p-value
Tumor Risk Grade	2600		
Low Risk		0.06 (-0.007,0.14)	
High Risk		0.09 (0.05,0.14)	0.519
Injection Site	6083		
Cervical		0.04 (0.02,0.05)	
Uterine		0.10 (0.04,0.15)	0.024
Dye Tracer	4015		
Indocyanine green (ICG)		0.04 (0.02,0.06)	
Blue dye		0.05 (0.006,0.09)	0.792
Tc-99m		0.12 (0.05,0.19)	0.042
Intraoperative Frozen Section	6145		
Yes		0.04 (0.02,0.06)	
No		0.04 (0.02,0.06)	0.984
Intraoperative Pathologic Ultrastaging	6095		
Yes		0.04 (0.02, 0.05)	
No		0.11 (0.04, 0.17)	0.036

### Comment

#### Principal findings

We performed a meta-analysis of 62 articles containing 6304 patients studying sentinel lymph nodes in endometrial cancer. We came to 3 main conclusions: 1. The overall false negative rate of sentinel lymph node biopsy for endometrial cancer was 4% (95% CL 3-5). 2. Cervical injection, indocyanine green dye tracer, and postoperative pathological ultrastaging reduced the false negative rate of SLNB. 3. There was no significant difference in the false negative rate between high-grade and low-grade patients (9% vs. 6%, p=0.519).

#### Comparison with existing literature

Sentinel lymph node biopsy (SLNB) has been widely accepted as the standard of care for surgical staging of low-grade endometrial cancer (EC), but its value in high-grade EC remains controversial. Various international guidelines suggest SLNB as a reasonable alternative option for high-grade EC subtypes (84, 85). However, although the value of SLNB in high-grade EC remains, there are questions about the accuracy of this technique in patients with high-grade histologic subtypes due to the greater risk of lymph node metastasis in high-risk endometrial cancer and concerns about isolated para-aortic lymph node involvement due to alternative lymphatic drainage (16). In a previous study, the results of a prospective single-center clinical study indicated that SLNB had a false negative rate of up to 80% in high-risk tissue types of endometrial cancer, while the false negative rate in low-risk types of endometrial cancer was only 0 (67). In another stud, it was concluded that SLN mapping was more effective in patients with LIR than in patients with HIR, with sensitivities of 100.00% and 75.00%, respectively (p > 0.05), and a higher rate of missed diagnoses in patients with HIR (82). In contrast, a prospective trial showed that SLN biopsy plus side-specific LND is a reasonable alternative to full LND when SLN is not detected in high-risk endometrial cancer (53). The results of a retrospective study support the same idea (86). The largest meta-analysis to date of false negative rates for sentinel lymph node biopsies for low- and high-risk endometrial cancer supported the conclusion that SLNB accurately detected lymph node metastases in high-grade EC with a false negative rate of 8% (95% CI 4-16), comparable to false negative rates for low-grade EC, melanoma, vulvar cancer, and breast cancer. This suggests that SLNB can replace complete lymph node dissection as the standard of care for surgical staging of patients with high-grade EC (17). The latest, a prospective cohort study (FIRES) included all risk groups, all histologic subtypes, and all stages of endometrial cancer, the accuracy of the sentinel lymph node in predicting lymph node metastasis is very high (87). In our meta-analysis, we also concluded that there was no significant difference in the false negative rate in patients with high-risk endometrial cancer compared with those with low-risk endometrial cancer. Unlike previous studies, our study involved an increased number of included articles as well as sample size, and we used meta-regression to explore the effect of tumor risk class on the combined false negative rate, which makes our findings more comprehensive and specific. This provides a close confirmation that sentinel lymph node biopsy is a feasible technical option in patients with high-risk endometrial cancer.

Currently, there is still controversy about the injection site and other modalities of SLN localization tracers. The injection modalities can be roughly divided into two main categories: cervical injection and uterine body injection; uterine body injection can be subclassified as subserosal tissue, deeper myometrial and hysteroscopically guided peritumoral injections. Considerable prospective data suggest that myometrial and endometrial injections appear to be more compatible with lymphatic drainage of endometrial tumors (88), but the complexity of their manipulation makes them not easily achievable. Cervical injections are technically more straightforward; however, their distance from the tumor raises concerns about their effectiveness (34). In contrast, proponents of cervical injection sites for endometrial cancer argue that the anatomical distribution of the SLNs corresponds to injections into the uterine corpus and coincides with the most common sites of lymphatic metastasis (internal iliac, external iliac, and obturator lymph nodes) for endometrial cancer (89). A prospective study showed that two lymphatic pathways consistent with pelvic SLNs were identified regardless of whether the injection site was the cervix or uterine fundus (90). This suggests that the location of the pelvic channel and SLNs is independent of the tracer injection site. Even with these controversies, due to the simplicity and time-saving nature of cervical injections compared to other injection modalities, the cervix is easily accessible even in minimally invasive procedures, and reliable injections can be performed with minimal additional equipment (91, 92). Therefore, cervical injections are used in most patients and currently represent the most reported modality in the literature. Most of the 62 articles in our study 42 (67.7%) involved use of only cervical injections, 12 (19.4%) involved use of only uterine injections, and the remaining 8 (12.9%) involved use of cervical and/or uterine injections. Some prospective and retrospective studies have demonstrated that pelvic sentinel lymph node localization by cervical tracer injection is a feasible and accurate technique for lymph node evaluation in endometrial cancer (52, 65, 90, 93). Especially for high-risk cancers, the cervix has been shown to be a viable and accurate site for tracer injection (92). However, it has been shown that cervical injections have a significantly lower para-aortic SLN detection rate than uterine injections (7% vs. 27%, p=0.001) (17). Uterine injections of para-abdominal aortic SNs test much higher (94). However, most studies have shown that cervical injections are associated with a significantly higher bilateral SLN detection rate than uterine injections, especially for the pelvic region. A large review of the literature on current techniques and outcomes of lymphatic localization of endometrial cancer summarized the detection rates of injection in the uterine corpus (7 studies), cervical injection (7 studies), and hysteroscopic injection (6 studies) and concluded that despite the controversy, cervical injection is associated with high detection rates (95). From another study, it was noted that although the detection rate of SLNs in the para-aortic region was slightly higher in patients receiving uterine injections, the difference relative to cervical injections was not statistically significant; instead, cervical injections allowed for better identification of lymph nodes in the pelvic region (96). It is now well established that cervical injections increase detection rates, and while high lymph node detection rates may allow for lower leakage of metastatic lymph nodes, there are few studies on the correlation between cervical injections and false negative rates. In this study, we were the first to compare the false negative rates of cervical injections and uterine injections using meta-analysis, with the conclusion that the false negative rate of SLNs was significantly lower for cervical injections than for uterine injections (4% vs. 10%, p=0.024) (6803 patients). This is consistent with previous studies supporting cervical injections and demonstrates that cervical injections not only increase the detection rate but also reduce the incidence of false negative rates.

Currently a commonly used tracer for SLNB of endometrial cancer included technetium colloid (Tc), blue dye, and indocyanine green (ICG). ICG is currently the most widely used NIR fluorescent dye, and many studies have shown its high sensitivity, specificity, and lymph node detection rate (65, 97). Most guidelines insist on surgeon experience for ICG technique, and it has been demonstrated that the diagnostic accuracy of SLN increases with surgical team experience (98). Blue dye is easy to use and cost-effectiveness. However, the blue dye can diffuse to parametrial area thus interfering with the discovery of regional SLN (99). Since radionuclide needs to be injected 1 day before surgery, it is not conducive to timely observation of sentinel lymph nodes, and the detected lymph nodes may not be the first stop for regional drainage, this condition also occurs frequently among doctors who are initially learning about SLNB, it may be more difficult to detect SLNs close to the cervix as the gamma-probe picks up high activity from the injection site (24). Our study concluded the false negative rate of SLN was significantly lower for indocyanine green than for Tc-99 m (4% vs. 12%, p=0.042) (4015 patients), this is in line with the previous conclusions.

The sentinel lymph node technique is now commonly accepted, but low-volume lymph node metastases occurring as micrometastases (MMs) and isolated tumor cells (ITCs) may be overlooked in routine evaluation when only routine pathology is performed intraoperatively (23). Even though IFS is used intraoperatively, its frequent diagnostic inaccuracy leads to an increased rate of missed diagnoses, which affects the prognosis of patients. Ultrastaging localization of sentinel lymph nodes has been shown to increase the detection of lymph node metastases, including occult low-volume metastases (23, 84). This reduces morbidity compared to systemic pelvic and para-aortic lymph node dissection and provides important prognostic information needed for adjuvant therapy (100). Ultrastaging techniques are essential for proper staging and reducing false negative rates (101). Ultrastaging can also be used as a complement to the deficiencies of frozen sections in endometrial cancer surgery, as an offset to diagnostic errors in IFS and to minimize their negative impact on patient care (102). Many studies have been designed to investigate the use of sentinel lymph node biopsy and ultrastaging techniques compared to lymph node dissection, especially for low-risk disease (46, 52, 103). Most scholars have concluded that low-volume metastases are not meaningful for adjuvant therapy and prognosis in low-risk endometrial cancer, whereas in high-risk endometrial cancer based on the objective effectiveness of adjuvant chemotherapy, it is speculated that adjuvant therapy guided by low-volume metastases may improve prognosis (104, 105). In a systematic review involving 15 studies and including 2259 patients, sentinel lymph nodes were examined using conventional hematoxylin and eosin staining. Subsequently, multiple ultrastaging methods were used, and 14% of patients were found to have positive sentinel lymph nodes. In 37% of these patients, these lymph nodes could be detected only by the ultrastaging method (106). It can also be argued that without pathologic ultrastaging, there is a high risk of missing micrometastases in lymph nodes or isolated tumor cells. In a large prospective FIRES study, the “ultrastaging” approach detected 54% of low-volume metastatic lesions in the sentinel lymph nodes, and it can be assumed that the false negative rate would be significantly higher if only conventional pathological examination was performed on the detected sentinel lymph nodes (52). Although the ultrastaging technique has many merits, serial sectioning is very time consuming for both technicians and pathologists (100). Additionally, its high price has led some hospitals to perform pathological ultrastaging on only some patients, and it is not universally available to every patient. Coupled with the fact that there are different types of ultrastaging techniques available, it is difficult to establish which one to use as a standard. At the same time, a prospective study noted that the OSNA method had high specificity and high accuracy in detecting SLN metastasis in apparent early-stage endometrial cancer (107). This requires us to explore the need for performing pathological ultrastaging. In our study, it was concluded that the use of postoperative pathological ultrastaging was associated with a lower rate of false negative SLNs (4% versus 11%, p=0.036) (6095 patients). This is in line with previous studies. It also demonstrates, once again, the need for the use of ultrastaging techniques in the biopsy of sentinel lymph nodes.

The current gold standard for the treatment of endometrial cancer is hysterectomy with bilateral salpingooophorectomy (BSO) with lymphadenectomy (108, 109). Nevertheless, in selected cases of patients desiring pregnancy, fertility-sparing treatment (FST) can be proposed, however, the status of lymph nodes during FST cannot be well investigated and evaluated (110). At present, imaging methods such as B-ultrasound, CT and MRI can also be used for the diagnosis of myometrial infiltration and lymph node metastasis (111–113). However, MRI examination is not popular due to its high cost and contraindications, B-ultrasound examination is low in accuracy, and CT examination is limited to endometrial lesions and has no diagnostic value. This makes FST a potential risk factor for false negative lymph node results.

## Strengths and limitations

Our study is the largest meta-analysis to date on sentinel lymph node biopsy for endometrial cancer and is the first to involve comprehensively performing stratified and meta-regression analyses of the effect of patient (tracer, injection site, tumor risk grade, intraoperative frozen section, and pathology ultrastaging) characteristics on combined false negative rates. Before us, the largest meta-analysis examining false negative rates for high-risk endometrial cancer included only nine articles (17). Our study has a huge advantage in terms of the number of articles compared to those in previous studies, from 9 previously to 23 in our study, which allows us to have a more comprehensive summary of high-risk endometrial cancers on the basis of our predecessors and a better response to the false negative rate. However, due to the relatively low incidence of lymph node metastasis in early-stage endometrial cancers, especially for low-risk endometrial cancers, false negative rates could not be obtained for many low-risk endometrial cancers because the statistical analyses were based on the number of lymph node-positive patients. This consideration led to the fact that only 7 of the 23 studies that we included provided reference to the false negative rates of low-risk endometrial cancers, whereas the majority of the articles reported false negative rates of high-risk endometrial cancers. The discrepancy in the number of the low-risk endometrial cancers in the comparison of false negative rates of two groups of low- and high-risk patients may have biased the results to some extent.

## Conclusions and implications

The current overall false negative rate for sentinel lymph node biopsy for endometrial cancer is 4% (95% CL 3-5). Sentinel lymph node biopsy for tracer injection in other parts of the uterus (other than the cervix), Tc-99m dye tracer, and failure to perform postoperative pathologic ultrastaging are risk factors for a high false negative rate of SLNB in patients with endometrial cancer; therefore, great attention should be given to the occurrence of leakage of lymph node metastasis after SLNB in this population. There is no difference in the false negative rate of sentinel lymph node biopsy in high-risk versus low-risk endometrial cancer patients, and performing sentinel lymph node biopsy in high-risk endometrial cancer patients is a viable technical option.

## Recommendations

In summary, the author provides recommendations for conducting future Sentinel lymph node biopsy of endometrial cancer. The research design should conform to the internationally recognized SPIRIT declaration, strictly follow the PICO principle, and register the research plan before the trial, and the final report should be standardized according to the CONSORT declaration.

## Author contributions

M-SF: Conceptualization, Data curation, Formal analysis, Investigation, Methodology, Project administration, Writing – original draft, Writing – review & editing. K-XQ: Writing – review & editing. D-YW: Writing – review & editing. HW: Writing – review & editing. W-WZ: Writing – review & editing. LY: Conceptualization, Funding acquisition, Supervision, Writing – review & editing.

## References

[1] Crosbie EJ Kitson SJ McAlpine JN Mukhopadhyay A Powell ME Singh N. Endometrial cancer. Lancet. (2022) 399:1412–28. doi: 10.1016/S0140-6736(22)00323-3 35397864

[2] Siegel R Naishadham D Jemal A. Cancer statistics 2023. CA Cancer J Clin. (2013) 63:11–30. doi: 10.3322/caac.21166 23335087

[3] Abu-Rustum NR. Sentinel lymph node mapping for endometrial cancer: a modern approach to surgical staging. J Natl Compr Canc Netw. (2014) 12:288–97. doi: 10.6004/jnccn.2014.0026 24586087

[4] William J Ling C Renata R Leslie H David E Barroilhet L Practice bulletin no. 149: endometrial cancer. Obstet Gynecol. (2015) 125:1006–26. doi: 10.1097/01.AOG.0000462977.61229.de 25798986

[5] Benedetti Panici P Basile S Maneschi F Alberto Lissoni A Signorelli M Scambia G. Systematic pelvic lymphadenectomy vs. no lymphadenectomy in early-stage endometrial carcinoma: randomized clinical trial. J Natl Cancer Inst. (2008) 100:1707–16. doi: 10.1093/jnci/djn397 19033573

[6] Kitchener H Swart AM Qian Q Amos C Parmar MK. Efficacy of systematic pelvic lymphadenectomy in endometrial cancer (MRC ASTEC trial): a randomised study. Lancet. (2009) 373:125–36. doi: 10.1016/s0140-6736(08)61766-3 PMC2646126 19070889

[7] Geppert B Lonnerfors C Bollino M Persson J. Sentinel lymph node biopsy in endometrial cancer-Feasibility, safety and lymphatic complications. Gynecol Oncol. (2018) 148:491–8. doi: 10.1016/j.ygyno.2017.12.017 29273307

[8] Restaino S Paglietti C Arcieri M Biasioli A Della Martina M Mariuzzi L. Management of patients diagnosed with endometrial cancer: comparison of guidelines. Cancers (Basel). (2023) 15(4):1091. doi: 10.3390/cancers15041091 36831434 PMC9954548

[9] Restaino S Buda A Puppo A Capozzi VA Sozzi G Casarin J. Anatomical distribution of sentinel lymph nodes in patients with endometrial cancer: a multicenter study. Int J Gynecol Cancer. (2022) 32:517–24. doi: 10.1136/ijgc-2021-003253 35110375

[10] Koskas M Amant F Mirza MR Creutzberg CL. Cancer of the corpus uteri: 2021 update. Int J Gynaecol Obstet. (2021) 155 Suppl 1:45–60. doi: 10.1002/ijgo.13866 34669196 PMC9297903

[11] Concin N Matias-Guiu X Vergote I Cibula D Mirza MR Marnitz S. ESGO/ESTRO/ESP guidelines for the management of patients with endometrial carcinoma. Int J Gynecol Cancer. (2021) 31:12–39. doi: 10.1136/ijgc-2020-002230 33397713

[12] How J Gauthier C Abitbol J Lau S Salvador S Gotlieb R. Impact of sentinel lymph node mapping on recurrence patterns in endometrial cancer. Gynecol Oncol. (2017) 144:503–9. doi: 10.1016/j.ygyno.2017.01.013 28104296

[13] Schlappe BA Weaver AL Ducie JA Eriksson AGZ Dowdy SC Cliby WA. Multicenter study comparing oncologic outcomes between two nodal assessment methods in patients with deeply invasive endometrioid endometrial carcinoma: A sentinel lymph node algorithm versus a comprehensive pelvic and paraaortic lymphadenectomy. Gynecol Oncol. (2018) 151:235–42. doi: 10.1016/j.ygyno.2018.08.022 PMC6214768 30177461

[14] Bogani G Murgia F Ditto A Raspagliesi F. Sentinel node mapping vs. lymphadenectomy in endometrial cancer: A systematic review and meta-analysis. Gynecol Oncol. (2019) 153:676–83. doi: 10.1016/j.ygyno.2019.03.254 30952370

[15] Baiocchi G Cemc Andrade R Moretti-Marques R Tsunoda AT Alvarenga-Bezerra V Lopes A. Sentinel lymph node mapping versus sentinel lymph node mapping with systematic lymphadenectomy in endometrial cancer: an open-label, non-inferiority, randomized trial (ALICE trial). Int J Gynecol Cancer. (2022) 32:676–9. doi: 10.1136/ijgc-2022-003378 35236752

[16] Salman L Cusimano MC Marchocki Z Ferguson SE. Sentinel lymph node mapping in high-grade endometrial cancer. Curr Oncol. (2022) 29:1123–35. doi: 10.3390/curroncol29020096 PMC8870608 35200595

[17] Marchocki Z Cusimano MC Clarfield L Kim SR Fazelzad R Espin-Garcia O. Sentinel lymph node biopsy in high-grade endometrial cancer: a systematic review and meta-analysis of performance characteristics. Am J Obstet Gynecol. (2021) 225:367.e1–367.e39. doi: 10.1016/j.ajog.2021.05.034 34058168

[18] Ballester M Dubernard G Lécuru F Heitz D Mathevet P Marret H. Detection rate and diagnostic accuracy of sentinel-node biopsy in early stage endometrial cancer: a prospective multicentre study (SENTI-ENDO). Lancet Oncol. (2011) 12:469–76. doi: 10.1016/s1470-2045(11)70070-5 21489874

[19] Ehrisman J Secord AA Berchuck A Lee PS Di Santo N Lopez-Acevedo M. Performance of sentinel lymph node biopsy in high-risk endometrial cancer. Gynecol Oncol Rep. (2016) 17:69–71. doi: 10.1016/j.gore.2016.04.002 27453926 PMC4941561

[20] Cusimano MC Vicus D Pulman K Maganti M Bernardini MQ Bouchard-Fortier G. Assessment of sentinel lymph node biopsy vs lymphadenectomy for intermediate- and high-grade endometrial cancer staging. JAMA Surg. (2021) 156:157–64. doi: 10.1001/jamasurg.2020.5060 PMC7658802 33175109

[21] Pineda VG Zapardiel I Gracia M Siegrist J Diestro MD Alonso M. Avoiding full lymphadenectomies in intermediate- and high-risk endometrial cancer by sentinel lymph node biopsy implementation. Front Oncol. (2021) 11:654285. doi: 10.3389/fonc.2021.654285 33937061 PMC8082098

[22] Cibula D Oonk MH Abu-Rustum NR. Sentinel lymph node biopsy in the management of gynecologic cancer. Curr Opin Obstet Gynecol. (2015) 27:66–72. doi: 10.1097/GCO.0000000000000133 25502426

[23] Kim CH Soslow RA Park KJ Barber EL Khoury-Collado F Barlin JN. Pathologic ultrastaging improves micrometastasis detection in sentinel lymph nodes during endometrial cancer staging. Int J Gynecol Cancer. (2013) 23:964–70. doi: 10.1097/IGC.0b013e3182954da8 PMC4079038 23694985

[24] Cormier B Rozenholc AT Gotlieb W Plante M Giede C. Sentinel lymph node procedure in endometrial cancer: A systematic review and proposal for standardization of future research. Gynecologic Oncol. (2015) 138(2):478–85. doi: 10.1016/j.ygyno.2015.05.039 26047592

[25] Baiocchi G Mantoan H Kumagai LY Goncalves BT Badiglian-Filho L de Oliveira Menezes AN. The impact of sentinel node-mapping in staging high-risk endometrial cancer. Ann Surg Oncol. (2017) 24(13):3981–7. doi: 10.1245/s10434-017-6132-8 29058141

[26] Barranger E Cortez A Grahek D Callard P Uzan S Darai E. Laparoscopic sentinel node procedure using a combination of patent blue and radiocolloid in women with endometrial cancer. Ann Surg Oncol. (2004) 11:344–9. doi: 10.1245/aso.2004.07.005 14993032

[27] Niikura H Okamura C Utsunomiya H Yoshinaga K Akahira J Ito K. Sentinel lymph node detection in patients with endometrial cancer. Gynecol Oncol. (2004) 92:669–74. doi: 10.1016/j.ygyno.2003.10.039 14766264

[28] Altgassen C Pagenstecher J Hornung D Diedrich K Hornemann A. A new approach to label sentinel nodes in endometrial cancer. Gynecol Oncol. (2007) 105:457–61. doi: 10.1016/j.ygyno.2007.01.021 17313975

[29] Lopes LA Nicolau SM Baracat FF Baracat EC Goncalves WJ Santos HV. Sentinel lymph node in endometrial cancer. Int J Gynecol Cancer. (2007) 17:1113–7. doi: 10.1111/j.1525-1438.2007.00909.x 17386045

[30] Bats AS Clement D Larousserie F Le Frere-Belda MA Pierquet-Ghazzar N Hignette C. Does sentinel node biopsy improve the management of endometrial cancer? Data from 43 patients. J Surg Oncol. (2008) 97:141–5. doi: 10.1002/jso.20857 18050286

[31] Barranger E Delpech Y Coutant C Dubernard G Uzan S Darai E. Laparoscopic sentinel node mapping using combined detection for endometrial cancer: a study of 33 cases–is it a promising technique. Am J Surg. (2009) 197:1–7. doi: 10.1016/j.amjsurg.2007.10.021 18558387

[32] How J Lau S Press J Ferenczy A Pelmus M Stern J. Accuracy of sentinel lymph node detection following intra-operative cervical injection for endometrial cancer: a prospective study. Gynecol Oncol. (2012) 127:332–7. doi: 10.1016/j.ygyno.2012.08.018 22910695

[33] Solima E Martinelli F Ditto A Maccauro M Carcangiu M Mariani L. Diagnostic accuracy of sentinel node in endometrial cancer by using hysteroscopic injection of radiolabeled tracer. Gynecol Oncol. (2012) 126:419–23. doi: 10.1016/j.ygyno.2012.05.025 22659192

[34] Rossi EC Jackson A Ivanova A Boggess JF. Detection of sentinel nodes for endometrial cancer with robotic assisted fluorescence imaging: cervical versus hysteroscopic injection. Int J Gynecol Cancer. (2013) 23:1704–11. doi: 10.1097/IGC.0b013e3182a616f6 24177256

[35] Torne A Pahisa J Vidal-Sicart S Martinez-Roman S Paredes P Puerto B. Transvaginal ultrasound-guided myometrial injection of radiotracer (TUMIR): a new method for sentinel lymph node detection in endometrial cancer. Gynecol Oncol. (2013) 128:88–94. doi: 10.1016/j.ygyno.2012.10.008 23085461

[36] Lopez-De la Manzanara Cano C Cordero Garcia JM Martin-Francisco C Pascual-Ramirez J Parra CP Cespedes Casas C. Sentinel lymph node detection using 99mTc combined with methylene blue cervical injection for endometrial cancer surgical management: a prospective study. Int J Gynecol Cancer. (2014) 24:1048–53. doi: 10.1097/IGC.0000000000000158 24927249

[37] Raimond E Ballester M Hudry D Bendifallah S Darai E Graesslin O. Impact of sentinel lymph node biopsy on the therapeutic management of early-stage endometrial cancer: Results of a retrospective multicenter study. Gynecol Oncol. (2014) 133:506–11. doi: 10.1016/j.ygyno.2014.03.019 24642092

[38] Farghali MM Allam IS Abdelazim IA El-Kady OS Rashed AR Gareer WY. Accuracy of sentinel node in detecting lymph node metastasis in primary endometrial carcinoma. Asian Pac J Cancer Prev. (2015) 16:6691–6. doi: 10.7314/apjcp.2015.16.15.6691 26434896

[39] Favero G Pfiffer T Ribeiro A Carvalho JP Baracat EC Mechsner S. Laparoscopic sentinel lymph node detection after hysteroscopic injection of technetium-99 in patients with endometrial cancer. Int J Gynecol Cancer. (2015) 25:423–30. doi: 10.1097/IGC.0000000000000387 25695546

[40] How J Gotlieb WH Press JZ Abitbol J Pelmus M Ferenczy A. Comparing indocyanine green, technetium, and blue dye for sentinel lymph node mapping in endometrial cancer. Gynecol Oncol. (2015) 137:436–42. doi: 10.1016/j.ygyno.2015.04.004 25870917

[41] Naoura I Canlorbe G Bendifallah S Ballester M Darai E. Relevance of sentinel lymph node procedure for patients with high-risk endometrial cancer. Gynecol Oncol. (2015) 136:60–4. doi: 10.1016/j.ygyno.2014.10.027 25449312

[42] Sawicki S Lass P Wydra D. Sentinel lymph node biopsy in endometrial cancer–comparison of 2 detection methods. Int J Gynecol Cancer. (2015) 25:1044–50. doi: 10.1097/IGC.0000000000000447 25853384

[43] Touhami O Trinh XB Gregoire J Sebastianelli A Renaud MC Grondin K. Is a more comprehensive surgery necessary in patients with uterine serous carcinoma. Int J Gynecol Cancer. (2015) 25:1266–70. doi: 10.1097/IGC.0000000000000488 26067862

[44] Valha P Kucera E Sak P Stepanek O Michal M. Intraoperative subserosal approach to label sentinel nodes in intermediate and high-risk endometrial cancer. Eur J Gynaecol Oncol. (2015) 36:643–6. 26775344

[45] Buda A Crivellaro C Elisei F Di Martino G Guerra L De Ponti E. Impact of indocyanine green for sentinel lymph node mapping in early stage endometrial and cervical cancer: comparison with conventional radiotracer (99m)Tc and/or blue dye. Ann Surg Oncol. (2016) 23:2183–91. doi: 10.1245/s10434-015-5022-1 PMC4889617 26714944

[46] Holloway RW Gupta S Stavitzski NM Zhu X Takimoto EL Gubbi A. Sentinel lymph node mapping with staging lymphadenectomy for patients with endometrial cancer increases the detection of metastasis. Gynecol Oncol. (2016) 141:206–10. doi: 10.1016/j.ygyno.2016.02.018 26905211

[47] Paley PJ Veljovich DS Press JZ Isacson C Pizer E Shah C. A prospective investigation of fluorescence imaging to detect sentinel lymph nodes at robotic-assisted endometrial cancer staging. Am J Obstet Gynecol. (2016) 215:117 e1–7. doi: 10.1016/j.ajog.2015.12.046 26743505

[48] Papadia A Imboden S Siegenthaler F Gasparri ML Mohr S Lanz S. Laparoscopic indocyanine green sentinel lymph node mapping in endometrial cancer. Ann Surg Oncol. (2016) 23:2206–11. doi: 10.1245/s10434-016-5090-x PMC4889624 26790667

[49] Biliatis I Thomakos N Koutroumpa I Haidopoulos D Sotiropoulou M Antsaklis A. Subserosal uterine injection of blue dye for the identification of the sentinel node in patients with endometrial cancer: a feasibility study. Arch Gynecol Obstet. (2017) 296:565–70. doi: 10.1007/s00404-017-4468-8 28744616

[50] Farzaneh F Moridi A Azizmohammadi Z Ansari JM Hosseini MS Arab M. Value of sentinel lymph node (SLN) mapping and biopsy using combined intracervical radiotracers and blue dye injections for endometrial cancer. Asian Pac J Cancer Prev. (2017) 18:431–5. doi: 10.22034/APJCP.2017.18.2.431 PMC5454739 28345826

[51] Holloway RW Ahmad S Kendrick JE Bigsby GE Brudie LA Ghurani GB. A prospective cohort study comparing colorimetric and fluorescent imaging for sentinel lymph node mapping in endometrial cancer. Ann Surg Oncol. (2017) 24:1972–9. doi: 10.1245/s10434-017-5825-3 28265777

[52] Rossi EC Kowalski LD Scalici J Cantrell L Schuler K Hanna RK. A comparison of sentinel lymph node biopsy to lymphadenectomy for endometrial cancer staging (FIRES trial): a multicentre, prospective, cohort study. Lancet Oncol. (2017) 18:384–92. doi: 10.1016/s1470-2045(17)30068-2 28159465

[53] Soliman PT Westin SN Dioun S Sun CC Euscher E Munsell MF. A prospective validation study of sentinel lymph node mapping for high-risk endometrial cancer. Gynecol Oncol. (2017) 146:234–9. doi: 10.1016/j.ygyno.2017.05.016 PMC5860676 28528918

[54] Tanner EJ Ojalvo L Stone RL Levinson K Temkin SM Murdock T. The utility of sentinel lymph node mapping in high-grade endometrial cancer. Int J Gynecol Cancer. (2017) 27:1416–21. doi: 10.1097/IGC.0000000000001047 30814241

[55] Taskin S Sukur YE Altin D Ersoz CC Turgay B Kankaya D. Laparoscopic near-infrared fluorescent imaging as an alternative option for sentinel lymph node mapping in endometrial cancer: A prospective study. Int J Surg. (2017) 47:13–7. doi: 10.1016/j.ijsu.2017.09.015 28919095

[56] Touhami O Gregoire J Renaud MC Sebastianelli A Plante M. Performance of sentinel lymph node (SLN) mapping in high-risk endometrial cancer. Gynecol Oncol. (2017) 147:549–53. doi: 10.1016/j.ygyno.2017.09.014 28942993

[57] Body N Gregoire J Renaud MC Sebastianelli A Grondin K Plante M. Tips and tricks to improve sentinel lymph node mapping with Indocyanin green in endometrial cancer. Gynecol Oncol. (2018) 150:267–73. doi: 10.1016/j.ygyno.2018.06.001 29909967

[58] Eoh KJ Lee YJ Kim HS Lee JY Nam EJ Kim S. Two-step sentinel lymph node mapping strategy in endometrial cancer staging using fluorescent imaging: A novel sentinel lymph node tracer injection procedure. Surg Oncol. (2018) 27:514–9. doi: 10.1016/j.suronc.2018.07.001 30217312

[59] Rajanbabu A Agarwal R. A prospective evaluation of the sentinel node mapping algorithm in endometrial cancer and correlation of its performance against endometrial cancer risk subtypes. Eur J Obstet Gynecol Reprod Biol. (2018) 224:77–80. doi: 10.1016/j.ejogrb.2018.03.017 29554604

[60] Shimada C Todo Y Yamazaki H Takeshita S Okamoto K Minobe S. A feasibility study of sentinel lymph node mapping by cervical injection of a tracer in Japanese women with early stage endometrial cancer. Taiwan J Obstet Gynecol. (2018) 57:541–5. doi: 10.1016/j.tjog.2018.06.012 30122575

[61] Tanaka T Terai Y Fujiwara S Tanaka Y Sasaki H Tsunetoh S. The detection of sentinel lymph nodes in laparoscopic surgery can eliminate systemic lymphadenectomy for patients with early stage endometrial cancer. Int J Clin Oncol. (2018) 23:305–13. doi: 10.1007/s10147-017-1196-9 PMC5882620 29098518

[62] Togami S Kawamura T Fukuda M Yanazume S Kamio M Kobayashi H. Prospective study of sentinel lymph node mapping for endometrial cancer. Int J Gynaecol Obstet. (2018) 143:313–8. doi: 10.1002/ijgo.12651 30125949

[63] Backes FJ Cohen D Salani R Cohn DE O’Malley DM Fanning E. Prospective clinical trial of robotic sentinel lymph node assessment with isosulfane blue (ISB) and indocyanine green (ICG) in endometrial cancer and the impact of ultrastaging (NCT01818739). Gynecol Oncol. (2019) 153:496–9. doi: 10.1016/j.ygyno.2019.03.252 31230614

[64] Kennard JA Stephens AJ Ahmad S Zhu X Singh C McKenzie ND. Sentinel lymph nodes (SLN) in endometrial cancer: The relationship between primary tumor histology, SLN metastasis size, and non-sentinel node metastasis. Gynecol Oncol. (2019) 154:53–9. doi: 10.1016/j.ygyno.2019.04.654 31027899

[65] Persson J Salehi S Bollino M Lönnerfors C Falconer H Geppert B. Pelvic Sentinel lymph node detection in High-Risk Endometrial Cancer (SHREC-trial)-the final step towards a paradigm shift in surgical staging. Eur J Cancer. (2019) 116:77–85. doi: 10.1016/j.ejca.2019.04.025 31181536

[66] Wang T Hu Y He Y Sun P Guo Z. A retrospective validation study of sentinel lymph node mapping for high-risk endometrial cancer. Arch Gynecol Obstet. (2019) 299:1429–35. doi: 10.1007/s00404-019-05085-0 PMC6475504 30747328

[67] Ye L Li S Lu W He Q Li Y Li B. A prospective study of sentinel lymph node mapping for endometrial cancer: is it effective in high-risk subtypes. Oncologist. (2019) 24:e1381–7. doi: 10.1634/theoncologist.2019-0113 PMC6975967 31270269

[68] Zuo J Wu LY Cheng M Bai P Lei CZ Li N. Comparison study of laparoscopic sentinel lymph node mapping in endometrial carcinoma using carbon nanoparticles and lymphatic pathway verification. J Minim Invasive Gynecol. (2019) 26:1125–32. doi: 10.1016/j.jmig.2018.11.002 30445188

[69] Gezer S Duman Ozturk S Hekimsoy T Vural C Isgoren S Yucesoy I. Cervical versus endometrial injection for sentinel lymph node detection in endometrial cancer: a randomized clinical trial. Int J Gynecol Cancer. (2020) 30:325–31. doi: 10.1136/ijgc-2019-000860 32029429

[70] Martinelli F Ditto A Bogani G Leone Roberti Maggiore U Signorelli M Chiappa V. Sentinel lymph node mapping in endometrial cancer: performance of hysteroscopic injection of tracers. Int J Gynecol Cancer. (2020) 30:332–8. doi: 10.1136/ijgc-2019-000930 31911536

[71] Renz M Marjon N Devereaux K Raghavan S Folkins AK Karam A. Immediate intraoperative sentinel lymph node analysis by frozen section is predictive of lymph node metastasis in endometrial cancer. J Robot Surg. (2020) 14:35–40. doi: 10.1007/s11701-019-00928-z 30687881

[72] Taskin S Altin D Vatansever D Tokgozoglu N Karabuk E Turan H. Sentinel lymph node biopsy in early stage endometrial cancer: a Turkish gynecologic oncology group study (TRSGO-SLN-001). Int J Gynecol Cancer. (2020) 30:299–304. doi: 10.1136/ijgc-2019-000847 31857440

[73] Anirudhan S,V Balasubramani L. A feasibility study of sentinel lymph node biopsy in endometrial cancer using technetium 99m nanocolloid. Indian J Surg Oncol. (2020) 11:699–704. doi: 10.1007/s13193-019-01020-6 33299284 PMC7714867

[74] Angeles MA Migliorelli F Vidal-Sicart S Saco A Ordi J Ros C. Paraaortic sentinel lymph node detection in intermediate and high-risk endometrial cancer by transvaginal ultrasound-guided myometrial injection of radiotracer (TUMIR). J Gynecol Oncol. (2021) 32:e52. doi: 10.3802/jgo.2021.32.e52 33908710 PMC8192237

[75] Curcio E Miller B Giglio A Akoluk A Erler B Bosscher J. Sentinel lymph node sampling in robot-assisted staging of endometrial cancer. South Med J. (2021) 114:680–5. doi: 10.14423/SMJ.0000000000001319 34729610

[76] Liang S Wang Z Chen J Yang X Liang X Sun X. Carbon nanoparticles combined with indocyanine green for sentinel lymph node detection in endometrial carcinoma. J Surg Oncol. (2021) 124:411–9. doi: 10.1002/jso.26518 34086291

[77] Sanchez-Izquierdo N Vidal-Sicart S Campos F Torne A Angeles MA Migliorelli F. Detection of the sentinel lymph node with hybrid tracer (ICG-[(99m)Tc]Tc-albumin nanocolloid) in intermediate- and high-risk endometrial cancer: a feasibility study. EJNMMI Res. (2021) 11:123. doi: 10.1186/s13550-021-00863-x 34905122 PMC8671586

[78] Somashekhar SP Arvind R Kumar CR Ahuja V Ashwin KR. Sentinel node mapping using indocyanine green and near-infrared fluorescence imaging technology for endometrial cancer: A prospective study using a surgical algorithm in Indian patients. J Minim Access Surg. (2021) 17:479–85. doi: 10.4103/jmas.JMAS_154_20 PMC8486055 33605932

[79] Wang Q Wang B Wang L Xue Y Shan W Luo X. The efficiency of a combined injection technique for sentinel lymph node mapping in intermediate-high-risk endometrial cancer. J Surg Oncol. (2021) 124:1551–60. doi: 10.1002/jso.26666 34496048

[80] Altin D Taskin S Ortac F Tokgozoglu N Vatansever D Guler AH. Diagnostic accuracy of sentinel node biopsy in non-endometrioid, high-grade and/or deep myoinvasive endometrial cancer: A Turkish gynecologic oncology group study (TRSGO-SLN-006). Gynecol Oncol. (2022) 164:492–7. doi: 10.1016/j.ygyno.2022.01.009 35033380

[81] Gedgaudaite M Sukovas A Paskauskas S Bartusevicius A Atstupenaite V Svedas E. The feasibility of sentinel lymph-node, mapped with indocyanine green, biopsy in endometrial cancer patients: A prospective study. Medicina (Kaunas). (2022) 58(6):712. doi: 10.3390/medicina58060712 35743975 PMC9227427

[82] Xue Y Shan WW Wang Q Wang C Luo XZ Chen XJ. Efficacy of sentinel lymph node mapping in endometrial cancer with low- or high-intermediate risk. J Surg Oncol. (2022) 125:256–63. doi: 10.1002/jso.26694 34569625

[83] Torrent A Amengual J Sampol CM Ruiz M Rioja J Matheu G. Sentinel lymph node biopsy in endometrial cancer: dual injection, dual tracer-A multidisciplinary exhaustive approach to nodal staging. Cancers (Basel). (2022) 14(4):929. doi: 10.3390/cancers14040929 35205676 PMC8870578

[84] Koh WJ Abu-Rustum NR Bean S Bradley K Campos SM Cho KR. Uterine neoplasms, version 1.2018, NCCN clinical practice guidelines in oncology. J Natl Compr Canc Netw. (2018) 16:170–99. doi: 10.6004/jnccn.2018.0006 29439178

[85] Concin N Matias-Guiu X Vergote I Cibula D Mirza MR Marnitz S. ESGO/ESTRO/ESP guidelines for the management of patients with endometrial carcinoma. Radiother Oncol. (2021) 154:327–53. doi: 10.1016/j.radonc.2020.11.018 33712263

[86] Papadia A Gasparri ML Radan AP Stämpfli CAL Rau TT Mueller MD. Retrospective validation of the laparoscopic ICG SLN mapping in patients with grade 3 endometrial cancer. J Cancer Res Clin Oncol. (2018) 144:1385–93. doi: 10.1007/s00432-018-2648-y PMC11813295 29691646

[87] Khemworapong K Jaishuen A Srichaikul P Inthasorn P Viriyapak B Achariyapota V. The fluorescence imaging for laparoscopic and laparotomic endometrial sentinel lymph node biopsy (FILLES) trial: Siriraj gynecologic sentinel node of endometrial cancer (SiGN-En) study. J Surg Oncol. (2024) 129:403–9. doi: 10.1002/jso.27486 37859537

[88] Touboul C Bentivegna E Uzan C Gouy S Pautier P Lhomme C. Sentinel lymph node in endometrial cancer: a review. Curr Oncol Rep. (2013) 15:559–65. doi: 10.1007/s11912-013-0345-1 24190831

[89] Abu-Rustum NR Khoury-Collado F Pandit-Taskar N Soslow RA Dao F Sonoda Y. Sentinel lymph node mapping for grade 1 endometrial cancer: is it the answer to the surgical staging dilemma? Gynecol Oncol. (2009) 113:163–9. doi: 10.1016/j.ygyno.2009.01.003 PMC3959736 19232699

[90] Geppert B Lönnerfors C Bollino M Arechvo A Persson J. A study on uterine lymphatic anatomy for standardization of pelvic sentinel lymph node detection in endometrial cancer. Gynecol Oncol. (2017) 145:256–61. doi: 10.1016/j.ygyno.2017.02.018 28196672

[91] Bogani G Raspagliesi F Leone Roberti Maggiore U Mariani A. Current landscape and future perspective of sentinel node mapping in endometrial cancer. J Gynecol Oncol. (2018) 29:e94. doi: 10.3802/jgo.2018.29.e94 30207102 PMC6189438

[92] Rossi EC. Current state of sentinel lymph nodes for women with endometrial cancer. Int J Gynecol Cancer. (2019) 29:613–21. doi: 10.1136/ijgc-2018-000075 30712017

[93] Barlin JN Khoury-Collado F Kim CH Leitao MM Jr. Chi DS Sonoda Y. The importance of applying a sentinel lymph node mapping algorithm in endometrial cancer staging: beyond removal of blue nodes. Gynecol Oncol. (2012) 125:531–5. doi: 10.1016/j.ygyno.2012.02.021 22366409

[94] Sahbai S Taran FA Fiz F Staebler A Becker S Solomayer E. Pericervical injection of 99mTc-nanocolloid is superior to peritumoral injection for sentinel lymph node detection of endometrial cancer in SPECT/CT. Clin Nucl Med. (2016) 41:927–32. doi: 10.1097/rlu.0000000000001414 27749429

[95] Khoury-Collado F Abu-Rustum NR. Lymphatic mapping in endometrial cancer: a literature review of current techniques and results. Int J Gynecol Cancer. (2008) 18:1163–8. doi: 10.1111/j.1525-1438.2007.01188.x 18217960

[96] Ditto A Casarin I Pinelli C Perrone AM Scollo P Martinelli F. Hysteroscopic versus cervical injection for sentinel node detection in endometrial cancer: A multicenter prospective randomised controlled trial from the Multicenter Italian Trials in Ovarian cancer (MITO) study group. Eur J Cancer. (2020) 140:1–10. doi: 10.1016/j.ejca.2020.08.030 33027722

[97] Bargon CA Huibers A Young-Afat DA Jansen BAM Borel-Rinkes IHM Lavalaye J. Sentinel lymph node mapping in breast cancer patients through fluorescent imaging using indocyanine green: the INFLUENCE trial. Ann Surg. (2022) 276:913–20. doi: 10.1097/sla.0000000000005633 35894448

[98] Torrent A Amengual J Sampol CM Ruiz M Rioja J Matheu G. Sentinel lymph node biopsy in endometrial cancer: dual injection, dual tracer—A multidisciplinary exhaustive approach to nodal staging. Cancers. (2022) 14(4):929. doi: 10.3390/cancers14040929 35205676 PMC8870578

[99] Du J Li Y Wang Q Batchu N Zou J Sun C. Sentinel lymph node mapping in gynecological oncology. Oncol Lett. (2017) 14:7669–75. doi: 10.3892/ol.2017.7219 PMC5755034 29344213

[100] Lang-Averous G Croce S Mery E Devouassoux-Shisheboran M. Sentinel lymph node processing in gynecological cancer histopathology and molecular biology. Chin Clin Oncol. (2021) 10:17. doi: 10.21037/cco-20-192 33440947

[101] Delpech Y Cortez A Coutant C Callard P Uzan S Darai E. The sentinel node concept in endometrial cancer: histopathologic validation by serial section and immunohistochemistry. Ann Oncol. (2007) 18:1799–803. doi: 10.1093/annonc/mdm334 17709801

[102] Blakely M Liu Y Rahaman J Prasad-Hayes M Tismenetsky M Wang X. Sentinel lymph node ultra-staging as a supplement for endometrial cancer intraoperative frozen section deficiencies. Int J Gynecol Pathol. (2019) 38:52–8. doi: 10.1097/PGP.0000000000000463 28968296

[103] Holloway RW Abu-Rustum NR Backes FJ Boggess JF Gotlieb WH Jeffrey Lowery W. Sentinel lymph node mapping and staging in endometrial cancer: A Society of Gynecologic Oncology literature review with consensus recommendations. Gynecol Oncol. (2017) 146:405–15. doi: 10.1016/j.ygyno.2017.05.027 PMC6075736 28566221

[104] St Clair CM Eriksson AG Ducie JA Jewell EL Alektiar KM Hensley ML. Low-volume lymph node metastasis discovered during sentinel lymph node mapping for endometrial carcinoma. Ann Surg Oncol. (2016) 23:1653–9. doi: 10.1245/s10434-015-5040-z PMC4835811 26714954

[105] Plante M Stanleigh J Renaud MC Sebastianelli A Grondin K Grégoire J. Isolated tumor cells identified by sentinel lymph node mapping in endometrial cancer: Does adjuvant treatment matter. Gynecol Oncol. (2017) 146:240–6. doi: 10.1016/j.ygyno.2017.05.024 28577885

[106] Burg LC Hengeveld EM In ‘t Hout J Bulten J Bult P Zusterzeel PLM. Ultrastaging methods of sentinel lymph nodes in endometrial cancer - a systematic review. Int J Gynecol Cancer. (2021) 31:744–53. doi: 10.1136/ijgc-2020-001964 33187974

[107] La Fera E Bizzarri N Petrecca A Monterossi G Dinoi G Zannoni GF. Evaluation of the one-step nucleic acid amplification method for rapid detection of lymph node metastases in endometrial cancer: prospective, multicenter, comparative study. Int J Gynecol Cancer. (2023) 33:1063–9. doi: 10.1136/ijgc-2023-004346 37105584

[108] Restaino S Ronsini C Finelli A Perrone E Scambia G Fanfani F. Role of blue dye for sentinel lymph node detection in early endometrial cancer. Gynecol Surg. (2017) 14:23. doi: 10.1186/s10397-017-1026-0 29213225 PMC5707225

[109] Gueli Alletti S Restaino S Finelli A Ronsini C Lucidi A Scambia G. Step by step total laparoscopic hysterectomy with uterine arteries ligation at the origin. J Minim Invasive Gynecol. (2020) 27:22–3. doi: 10.1016/j.jmig.2019.06.001 31201941

[110] Ronsini C Mosca L Iavarone I Nicoletti R Vinci D Carotenuto RM. Oncological outcomes in fertility-sparing treatment in stage IA-G2 endometrial cancer. Front Oncol. (2022) 12:965029. doi: 10.3389/fonc.2022.965029 36185260 PMC9524219

[111] Hosoi A Ueda Y Shindo M Nakagawa S Matsuzaki S Kobayashi E. Endometrial thickness measured by ultrasonography in postmenopausal patients with endometrial carcinoma has significance, irrespective of histological subtype. Int J Gynecol Cancer. (2013) 23:1266–9. doi: 10.1097/IGC.0b013e31829f1857 23851678

[112] Koplay M Dogan NU Erdogan H Sivri M Erol C Nayman A. Diagnostic efficacy of diffusion-weighted MRI for pre-operative assessment of myometrial and cervical invasion and pelvic lymph node metastasis in endometrial carcinoma. J Med Imaging Radiat Oncol. (2014) 58:538–46. doi: 10.1111/1754-9485.12209 25046775

[113] Wan Q Jiao Q Li X Zhou J Zou Q Deng Y. Value of (18)F-FDG PET/CT and MRI in diagnosing primary endometrial small cell carcinoma. Chin J Cancer Res. (2014) 26:627–31. doi: 10.3978/j.issn.1000-9604.2014.10.04 PMC4220262 25400430

